# Stem Cell-Derived β Cells: A Versatile Research Platform to Interrogate the Genetic Basis of β Cell Dysfunction

**DOI:** 10.3390/ijms23010501

**Published:** 2022-01-02

**Authors:** Alberto Bartolomé

**Affiliations:** Instituto de Investigaciones Biomédicas Alberto Sols, CSIC-UAM, 28029 Madrid, Spain; abartolome@iib.uam.es

**Keywords:** diabetes, T1D, T2D, genetic variants, SNP, GWAS, beta cell, stem cell, iPSC, hESC

## Abstract

Pancreatic β cell dysfunction is a central component of diabetes progression. During the last decades, the genetic basis of several monogenic forms of diabetes has been recognized. Genome-wide association studies (GWAS) have also facilitated the identification of common genetic variants associated with an increased risk of diabetes. These studies highlight the importance of impaired β cell function in all forms of diabetes. However, how most of these risk variants confer disease risk, remains unanswered. Understanding the specific contribution of genetic variants and the precise role of their molecular effectors is the next step toward developing treatments that target β cell dysfunction in the era of personalized medicine. Protocols that allow derivation of β cells from pluripotent stem cells, represent a powerful research tool that allows modeling of human development and versatile experimental designs that can be used to shed some light on diabetes pathophysiology. This article reviews different models to study the genetic basis of β cell dysfunction, focusing on the recent advances made possible by stem cell applications in the field of diabetes research.

## 1. Introduction

Pancreatic β cells produce insulin, an anabolic hormone that regulates key metabolic functions. Blood glucose levels, the primary systemic energy currency, are tightly controlled by insulin. This is enabled by the glucose-sensing capabilities of β cells and the secretion of insulin as a function of the organism’s nutritional status. Loss of β cell function results in elevated blood glucose levels, which is the defining feature of the heterogeneous metabolic disorder termed “diabetes mellitus”. Over 451 million adults live with diabetes worldwide, with estimates of these numbers to be increased to 693 million by 2045 [1]. The magnitude of these numbers imposes a burden on global health and healthcare systems, making the fight against the disease of prime importance. Diabetes has been historically classified into the following categories: type 1 diabetes (T1D), characterized by insulin deficiency as a result of autoimmune β cell destruction [2]; type 2 diabetes (T2D), defined by insulin resistance but with clinical manifestation only after inadequate β cell compensation [3]. Besides these major two types, there is a collection of inherited disorders termed MODY (mature onset diabetes of the young) [4] and other forms that manifest soon after birth (neonatal diabetes) [5]; all of these caused by variations of a single gene that results in β cell dysfunction. This oversimplistic view of classifying subgroups of diabetes as “polygenic” or “monogenic” disorders is becoming less distinct in light of new advances in human disease genetics [6]. Studies are now suggesting the role of rare variants in susceptibility to common diseases [7,8], including diabetes [9,10]. For instance, variants classically associated with MODY are also implicated in autoimmune diabetes [11] and T2D development [9,12,13]. The importance of the monogenic diabetes-driver genes is patent in the course of diabetes progression [14], as expression of the “MODY network” is critically altered in T2D human β cells [15].

While the individual risk of diabetes is influenced by genetic factors [16,17]—object of interest for this review—we should not forget that environmental factors and lifestyle largely contribute to disease development, with global changes in diet and sedentary behaviors being the drivers of the epidemic proportions of the disease [18,19,20]. This review discusses the various models used to uncover and understand the genetic modifiers of β cell function, focusing on recent technological advances in the use of human stem cell differentiation protocols. I review some of the latest applications of these techniques and discuss the different categories of genetic modifiers and altered processes that are associated with β cell dysfunction. Finally, I address some of the current limitations of stem cell-based research strategies.

## 2. Models to Study the Genetic Basis of β Cell Dysfunction

### 2.1. Animal Models

Comparative biology has been fundamental to understanding diabetes pathophysiology in humans. From Minkowski’s seminal discoveries of the “glycemia-lowering” function of the pancreas in 1890 [21] to the use of a wide array of monogenic mouse models, nearly all milestones in diabetes research have involved animal research. These scientific endeavors have allowed building a corpus of phenotypic data, enhancing our understanding of many aspects of the molecular biology of diabetes [22]. Specific mouse lines, tailored to investigate problems relevant for the genetics of human diabetes, have shed some light on the biology of these. Examples include research into T2D-GWAS identified risk loci using mouse models: from *CDKN1C-KCNQ1* imprinted locus [23] to the generation of mice with a single missense mutation to model rare cases of monogenic diabetes (*MAFA*-S64F) [24]. Gene loss- or gain-of-function mouse models are the gold standard for in vivo functional validation [25]. Consortium-led research efforts have created genome-wide libraries for conditional knockout in C57BL/6N mouse embryonic stem cells (ESCs) [26,27]. Phenotyping of 449 knockout mouse lines derived from these—many of genes with no previously described role—uncovered 50 loci associated with diabetes and metabolic disorders [28].

Animal models continue to be a powerful tool for interrogating the genetic components of diabetes. Panels of recombinant inbred (RI) strains are a viable strategy to uncover new loci implicated in glucose metabolism. The Hybrid Mouse Diversity Panel (HMDP) consists of a panel of 100 strains (29 inbred and 71 RI strains) [29,30]. Examination of insulin resistance traits found genotype-related differences, and identified 15 significant loci associated with insulin resistance [31]. A phenotyping study of 40 different BXD mice strains (C57BL/6 × DBA)—tailored to study metabolism traits—uncovered *Dhtkd* as a regulator of 2-aminoadipate [32,33], a biomarker for diabetes risk [34]. This is a perfect example of how findings in mouse models can be translated to diabetes in humans. In fact, only recently a T2D-risk single nucleotide polymorphism (SNP) in an intergenic enhancer sequence in the *DHKTD1-CAMK1D* locus has been identified [35,36,37]. Similar approaches have been applied to investigate the genetic basis of T1D, with several congenic strains derived from the non-obese diabetic (NOD) mice, reviewed in [38]. Beyond mice other organisms, such as zebrafish [39,40] or Drosophila [41], have also proven useful for interrogating the genetics of diabetes.

### 2.2. Non-Human Cell Lines

In vitro studies using β cell lines are often a viable alternative to study certain aspects of β cell biology given the limited availability of pancreatic endocrine tissue. Cell lines derived from murine insulinoma have been widely used in research [42]. The most popular lines are MIN6 [43] and INS1/INS1E [44,45]. These cells have been used in unbiased drug screenings, monitoring insulin promoter activity and/or secretion [46,47,48], functional genomic screenings with targeted [49,50], genome-wide RNAi approaches [51] or genome-wide CRISPR screening [52]. A recent study using the NOD mice-derived insulinoma line (NIT-1) uncovered novel genes impacting T1D progression. In this study, NIT-1 cells transduced with a genome-wide CRISPR knockout library were transplanted into immunodeficient NOD mice, followed by splenocyte injection from diabetic mice to trigger β cell destruction. Surviving NIT-1 cells were analyzed to identify target genes that might mediate enhanced survival, and this led to *Rnls* identification as a mediator of autoimmune β cell death [53].

While studies in animals and cell lines derived from them are important to advance diabetes research, there are fundamental differences that limit the translatability of findings in these models, including intra-species variability and difficulties of recapitulating phenotypes observed in human disease [54,55]. For instance, while the systems and signaling pathways that are important for diabetes physiopathology have ancient evolutionary roots, the genetic variants influencing disease risk are very young, with species-specific origins [56,57]. Therefore, human β cell studies are a much-needed complement to the studies described above.

### 2.3. Human Cell Lines

Since 2011, a human β cell line is available for the scientific community (EndoC-βH1 and derivatives) [58,59]. These cells share electrophysiologic features with native human β cells [60], with comparative multi-omic data readily available [61]. EndoC-βH1 have been used in drug screening studies [62] as well as genome-wide CRISPR screening to identify genes that alter insulin content [63] or β cell survival in response to cytokines [64]. Derivatives of this line—with particular interest for screening studies—include a dual reporter of calcium flux and insulin secretion [65] and EndoC-βH2/3 for conditional excision of immortalizing transgenes [66,67]. It is important to note that EndoC-βH are transformed cells that display karyotypic aberrations [61], which warrants caution when considering them to study the genetics of human diabetes. Although EndoC-βH represent a significant advancement, they share fundamental differences with primary β cells. For this reason, studies on human islets from cadaveric donors are still the preferred approach for functional assays.

### 2.4. Human Islets

Studies in human islets are limited by the scarcity of donors and isolation centers. These studies are expensive and sometimes face additional problems such as differences in viability, purity and inherent functional heterogeneity that requires a high number of donors [68]. Many of the advances seen in β cell research during the last decade have relied on islets from donors. Unbiased -omic studies have explored the shape of the β cell genome identifying non-coding RNAs modulated in T2D [69], RNA splicing [70], regulatory elements [71], chromatin accessibility, DNA methylation [72,73,74] and three-dimensional chromatin architecture [75], findings that were integrated with GWAS signals. While large databases that integrate genomic information with tissue specific expression are a precious asset in identifying expression quantitative trait loci (eQTLs), these databases rarely contain information from pancreatic islets [76]. Several studies using genomic and islet transcriptomic data from hundreds of islet donors are filling this gap [77,78,79,80]. An explosion of studies in human islets at the single-cell level have documented the β cell heterogeneity, identifying altered pathways in T2D [81,82,83,84,85,86], and even associated single-cell transcriptomics with functional analysis through patch-clamp followed by single-cell RNA sequencing (scRNA-seq) [87]. Beyond cost and availability, functional genomic screenings in primary β cells are complicated [88]. Despite this, there are examples of targeted screenings such as RNAi of cell cycle components for human β cell proliferation [89] and CRISPR-mediated gain-of-function of master regulators identified in scRNA-seq [15].

### 2.5. Human Stem Cell-Derived β Cells

**Figure 1:**
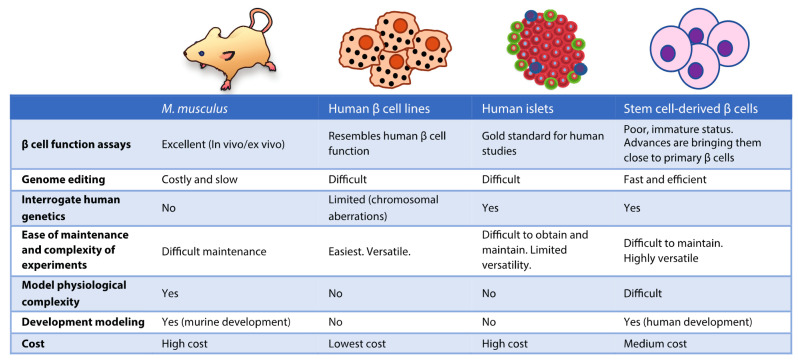
Comparison of model systems used in β cell research. Some of the advantages and limitations are summarized. Original illustrations except for vectorized laboratory mouse (CC BY-SA 3.0 license, David Liao).

The use of stem cells in research has the potential to revolutionize our understanding of human biology. Protocols to derive specific cell lineages—which can be of extreme scarcity—are emerging across all biomedical fields [90]. These protocols have an additional advantage, which is the possibility of modeling human development, something previously unattainable with the use of human biological samples (Figure 1). Stem cells have the ability to maintain their stemness through self-renewal and the potential of differentiating into diverse mature cell types. Stem cells can be classified into different categories: (i) embryonic stem cells (ESCs), which can be derived from the early embryo [91]; (ii) induced pluripotent stem cells (iPSCs), which allows somatic cells to be reprogrammed to an embryonic-like state by either expression of some defined factors [92] or by nuclear transfer into an oocyte, followed by derivation of stem cells [93]; and (iii) adult stem cells, which are undifferentiated cells that can be found in different tissues, with the potential of giving rise to different cell lineages [94].

Protocols for generating β cells from stem cells were built upon a large body of previous work on developmental biology, and have tried to mimic normal pancreas development through the use of various growth factors, specific inhibitors and other signaling molecules. Key stages of pancreas development include definitive endoderm (DE), primitive gut tube (PG), posterior foregut (PF), pancreatic progenitor (PP) and endocrine progenitor (EP) stages. The first ESC differentiation protocols to generate DE [95], were soon followed by others leading to the PP stage and hormone-producing endocrine cells [96]. These first protocols gave rise to polyhormonal endocrine cells, with poor glucose responsiveness. Transplantation of ESC-derived PPs into mice was far superior to in vitro differentiation, with respect to obtaining glucose-responsive stem cell-derived β cells (SC-β) [97,98,99]. Currently, there are ongoing clinical trials based on the use of encapsulated hESC-derived PPs as cell replacement therapy for T1D, with first-in-human phase 1/2 results already disseminated [100,101].

Other protocols (derived from those previously mentioned) successfully generated monohormonal SC-β entirely in vitro [102,103] but still with poor glucose responsiveness. A milestone in the field involved the generation of SC-β from patient fibroblast-derived iPSCs [104,105], also achieved by somatic cell nuclear transfer-derived ESCs [106]. This opened the door to autologous β cell replacement therapies.

Recent protocols have achieved further success in generating glucose-responsive SC-β by adjusting the last steps of the differentiation procedure and fine-tuning the cell clustering process [107,108]. Single-cell transcriptomics and functional profiling throughout the differentiation process have been key in identifying novel SC-β maturity markers and areas for protocol improvement, which will surely lead to better protocols in the upcoming years [109,110,111,112,113].

Isogenic hESC platforms are an attractive approach for functional studies. hESC can be engineered to incorporate custom reporters, transcriptional transactivators or gene-editing tools. HES3 and MEL1 hESC lines encoding a copy of *GFP* in the *INS* locus (*INS^GFP/wt^*) are useful to trace INS+ cells through the differentiation process [114]. hESC expressing luciferase (*GAPDH^luc/wt^*) can be used to monitor in vivo transplanted cells and organoids [115]. Some of the examples generated to date include inducible Cas9 for CRISPR screenings [116] and a transcriptional transactivator for gain-of-function studies [117].

The rest of this review focuses on the genetics of β cell dysfunction and stem cell-derived applications used to interrogate the genetic components of diabetes.

## 3. Genetic Basis of β Cell Dysfunction

**Figure 2:**
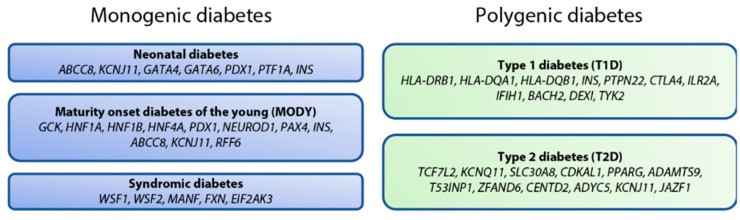
Summary of some of the genes involved in monogenic diabetes, and a small sample of risk loci identified for polygenic diabetes (>50 for T1D, >500 for T2D).

One of the most radical changes in the T2D field over the last two decades has been the ample recognition of the β cell as a key player in disease progression, as opposed to considering T2D as largely a “disease of insulin resistance”. This paradigm shift has come about in part due to our better understanding of the genetics of diabetes. T2D is a polygenic disease, but monogenic forms of diabetes are predominantly associated with genes that critically control β cell development and/or mature β cell function [118]. As mentioned in the introduction, “monogenic” and “polygenic” disorders are becoming less distinct [6], but for the sake of convenience and clarity, this section follows the standard classification (Figure 2), covering the use of stem cell-based protocols to investigate the genetic causes and biological mechanisms of disease.

### 3.1. Monogenic Diabetes

There is a wide array of diabetes caused by mutations in a single gene, which are generally autosomal dominant mutations that disrupt the coding sequence of proteins critical for β cell development and/or function. According to the time of diabetes onset, we can subdivide these between neonatal diabetes and maturity-onset diabetes of the young (MODY). Neonatal diabetes is mostly diagnosed during the first 6 months of life and can be transient or permanent depending on the degree of deleteriousness associated with the genetic variation [5]. The most common causes are alterations in the 6q24 imprinted locus [119], and activating mutations in the subunits that encode for the ATP-sensitive K^+^ channels (K_ATP_), *ABCC8* [120] or *KCNJ11* [121]. Mutations in critical regions of the *INS* gene, leading to poor proinsulin folding, ER stress and cell death, cause permanent neonatal diabetes [122]. Similar effects have been extensively studied in the Akita (Ins2-C96Y) [123] and Munich (Ins2-C95S) mouse models [124]. Rare mutations in *GATA4*, *GATA6*, *PDX1*, *PTF1A* or *ZNF808*, which cause pancreas agenesis, course with permanent neonatal diabetes [125,126,127,128].

MODY represents 1–2% of all diabetes, and is considered largely underdiagnosed [129,130]. MODY is a clinically heterogeneous group, with mutations in more than 15 genes described to date. The most commonly found cases arise from mutations in *GCK* [131], *HNF4A* [132], *HNF1A* [133] and *HNF1B* [134], accounting for up to 80% of all diagnosed MODY. Rarer cases can be found by mutations in *PDX1*, *NEUROD1*, *PAX4*, *INS*, *ABCC8*, *KCNJ11* and *RFX6*, in addition to other even rarer candidates: *KLF11*, *BLK*, *CEL* and *APPL1* [135]. A recent study identified mutations in *ONECUT1* as a novel MODY causative gene [136].

A third group of monogenic diabetes results from multiorgan syndromes. In these, diabetes can result as a consequence of peripheral defects, β cell dysfunction or a combination. Examples include *WFS1/WFS2* mutations in Wolfram-syndrome [137], *MANF* mutations associated with a neurodevelopmental disorder [138], repeat insertions in *FXN* causing Friedreich’s ataxia [139] and *EIF2AK3* mutations in Wolcott-Rallison syndrome [140].

Stem cells are an invaluable tool for investigating the molecular mechanisms of monogenic diabetes and hold the promise of a potential curative treatment [141]. Studies using human islets from monogenic diabetes patients are not a real possibility since these cases only represent a small fraction of all diabetes cases. iPSCs from patients and mutation-corrected isogenic controls are probably the best available tool for modeling human monogenic diseases. Isogenic controls can alternatively be generated by the introduction of the mutation of interest in iPSCs from healthy individuals or well-characterized ESC lines. Many studies describe the use of lines derived from patients and healthy family controls, which is a suboptimal approach [142]. The accelerated development of gene editing technologies with the CRISPR/Cas9 toolbox now enables the use of isogenic controls. Several patient-derived iPSC lines to study monogenic diabetes have been reported up to date, covering many of the genes previously mentioned. While many of the lines remain uncharacterized to date, some of these have been subjected to differentiation protocols and allowed to describe mechanisms underlying disease. The lines and studies are described below.

- *HNF1A*

iPSCs from patients are described in [143,144,145,146,147]. Cardenas-Diaz and colleagues studied *HNF1A* −/− and +/− lines generated from hESCs and subjected these to β cell differentiation protocols. They found lower expression of *PAX4* in *HNF1A* deficient lines, shifting the gene expression signature toward α cells [148]. González and colleagues studied both *HNF1A*-deficient lines generated from hESCs but also generated iPSC lines from patients and mutation-corrected isogenic controls. They also found a shift toward α cell fate, impaired GSIS, accumulation of abnormal insulin granules and described a sub-stoichiometric insulin:C-peptide secretion in *HNF1A* hypomorphic SC-β cells [146].

- *HNF4A*

Patient-derived iPSCs are described in [144,149,150,151]. Some studies have performed transcriptional profiling of *HNF4A*-mutant lines in the developmental stages of PG and PP [149,150], as well as proteomics at the SC-β stage [151]. These studies found marked alterations in *HNF4A*-mutant lines, such as decreased *HNF1A* and *PDX1* expression during differentiation or upregulation of the Wnt signaling pathway at the SC-β stage.

- *HNF1B*

Haploinsufficient *Hnf1b* mice do not phenocopy the effects seen in human patients [152], which highlights the need for human modeling of the disease. iPSC lines from patients are described in [144,153]. Hypomorphic *HNF1B* cells displayed altered differentiation, with a compensatory increase in many transcription factors associated with early pancreas development and a marked decrease of *PAX6* expression [154].

Other described iPSC lines were generated from patients with mutations in *GCK* [144], *PDX1* [13,155], *KCNJ11* [156], *INS* [157,158], *YIPF5* [159], *GATA6* [160], *WFS1* [161,162], *TRMT10A* [163], *ONECUT* [136], *FOXA2* [164] and activating mutations in *STAT3* [165]. Patient-derived iPSCs were also used to model congenital hyperinsulinism caused by *ABCC8* deficiency [166].

In addition, other studies in isogenic hESC platforms have explored the role of some genes known to be important for pancreas development, associated with diabetes in rare hereditary syndromes. The use of an hESC to study the role of some of these genes is particularly interesting since mouse models cannot always recapitulate the phenotypes observed in humans [167]. *GATA4*- and *GATA6*-deficient hESC lines were found to impact DE and PP stages. Additionally, *GATA6* haploinsufficiency impairs the expression of pro-endocrine markers during the PP stage [168]. Deletion of *NEUROD1* in hESCs with subsequent differentiation into SC-β results in a lower number of insulin-positive cells, which is associated with reduced expression of key β cell transcription factors [169]. Likewise, *GLIS3*-deficient hESCs show impaired β cell differentiation and increased cell death at the EP and SC-β stages [170]. Zhu and colleagues described the role of several factors by using gain- and loss-of-function strategies [117]. NOTCH1 and NEUROG3 gain-of-function during the PP stage have opposing effects in endocrine development by blocking or promoting it, respectively, which fits well with our knowledge of murine pancreas development [171,172]. Neurog3 is absolutely required for endocrine pancreas development in mice, but *NEUROG3* −/− hESCs still have a residual capacity of generating hormone-expressing cells [117], which fits with reports from patients with homozygous mutations [173,174]. On the other hand, another study using *NEUROG3* −/− hESCs found an absolute requirement for endocrine lineage development, and determined that only 10% of NEUROG3 is required for generation of endocrine cells, arguing that hypomorphic human alleles might retain some residual activity that explains some of the observed differences [175].

These examples highlight the interest in conducting studies involving mutations observed in human patients. Lessons learned from disease modeling can quickly lead to therapeutic advances in the era of personalized medicine. A precedent of rapid “bench to bedside” development occurred in neonatal diabetes. Soon after the discovery of K_ATP_ [176], and identification of patients with gain-of-function mutations [121], sulfonylureas proved to be a superior alternative to insulin treatment for this subgroup of neonatal diabetes patients [177].

### 3.2. Type 2 Diabetes (T2D)

Over the last years, consortium-driven genome-wide association studies (GWAS) have successfully identified common variants at genomic loci associated with an increased risk of T2D [36,178,179,180,181,182,183,184,185,186,187,188,189]. A few of the coding variants identified in these studies result in hypomorphic variants [9,190]. Still, most of the single-nucleotide polymorphisms (SNPs) identified in T2D GWAS map to non-coding regions of the genome (intronic or intergenic). There has been some progress elucidating the molecular mechanisms underlying some of these variants, but most remain unexplored. The multidimensional nature of the biology behind these associations makes further study of these signals a fairly complicated task: (1) Strong signals of association found in non-coding regions are often non-causal. Tag-SNPs used in GWAS microarrays are surrogates for large genomic regions [191], and disease-risk association might result from linkage disequilibrium (LD) with another causal variant [192,193] or depend on multiple variants acting in coordination [194]. Patterns of LD between SNPs can be complex, hindering the identification of causal regulatory variants [195]. (2) GWAS variants are frequently found associated with gene expression effects in nearby genes. Such abundance of associations complicates follow-up studies [196]. The magnitude, and in some cases the direction, of regulatory variants in expression quantitative trait loci (eQTL) effects differs among tissues [197,198,199]. In many instances, reported differences in gene expression might be the consequence and not the cause of disease association [200]. (3) The “variable of time” is often ignored in eQTL studies and databases. Gene expression differences may be relevant for a given phenotype, but in some circumstances, this might only be detected during a brief window of time. Some of the best examples of this lie with the potential effects on β cell biology (e.g., during a specific stage in pancreas development or when adult β cells are challenged).

The heritability of complex traits such as T2D is associated with a large number of variants, each of them with small effects [201,202]. By computing the sum of the effects of individual risk alleles on the phenotype of interest, a “polygenic risk score” can be calculated [202,203,204]. This score has practical applications, such as advising more frequent screenings in at-risk patients or guiding recommendations in behavioral modifications aimed at reducing disease risk. Still, the full potential application of the knowledge derived from GWAS and follow-up studies lies beyond these risk scores [203]. Characterization of the biology behind genetic variants and risk association for each of the identified loci could enable a qualitative stratification of patients for a refined treatment. Diagnosis and treatment of heterogeneous diseases such as T2D can potentially be transformed in the era of personalized medicine [205]. For example, patients could be classified according to a refined genetic score on β cell dysfunction, insulin resistance, cardiovascular risk or even scores that point to individual signaling pathways (GLP1, PPARG, etc.). However, to harness the full potential of personalized medicine, basic research is undoubtedly needed to disentangle the biology of single genetic variants. For this, stem cell research can provide unique tools, and there have been several approaches headed this way, which are summarized below and in Table 1.

- *CDKAL1*

The CDK5 regulatory associated protein 1-like 1 (CDKAL1) is a methylthiotransferase that is required for modification of tRNA^Lys^ and the accurate translation of AAA and AAG codons [206]. T2D-risk variants in the *CDKAL1* locus were identified in the first wave of T2D-GWAS [187]. Risk variants are associated with *CDKAL1* impaired splicing, and miRNA-dependent transcript decay [207]. β cell-specific *Cdkal1* KO mice display glucose intolerance and impaired insulin secretion, along with elevated markers of ER stress [206]. Zeng and colleagues used an isogenic hESC platform to investigate GWAS-identified T2D susceptibility loci, including *CDKAL1.* Differentiation of *CDKAL1* deficient hESCs toward SC-β was not altered, but SC-β showed impaired glucose-stimulated insulin secretion (GSIS), although KCl or arginine-induced secretion was not impacted [208]. A follow-up study using *CDKAL1*-deficient SC-β found decreased expression of the metallothionein gene family, higher susceptibility to ER stress and increased cell death [209].

- *KCNJ11*

This gene encodes for the Kir6.2 subunit of the K_ATP_ channel. Gain-of-function mutations are associated with neonatal diabetes [121], while loss-of-function results in congenital hyperinsulinism [210]. SNPs in the *KCNJ11* locus have been associated with T2D-risk in some populations [211,212]. SC-β derived from *KCNJ11* −*/*− hESCs show unaltered differentiation, but impaired insulin secretion [208]. Although *Kcnj11 +/*− mice display hyperinsulinism, homozygous loss of the gene results only in transient hyperinsulinemia in neonates, unexpectedly followed by loss of insulin secretion and glucose intolerance in adulthood [213]. Unfortunately, there are no studies on SC-β derived from *KCNJ11 +/*− hESCs or harboring patient-specific mutations.

- *KCNQ1*

This gene encodes a voltage-gated K^+^ channel, which is expressed in β cells, but with an unclear role in insulin secretion. *KCNQ1* missense mutations are associated with different congenital arrhythmia syndromes [214,215], with no reported effect on glucose homeostasis. Islets from *Kcnq1* −*/*− mice do not display defects in insulin secretion [23]. *KCNQ1* is an imprinted locus, and T2D-associated SNPs located in non-coding regions of the locus have been identified [216] and are more significantly correlated with T2D in Asian populations [217]. Interestingly, T2D association depends on the parental origin of the risk allele [218]. There are conflicting reports on the consequences of *KCNQ1* gain- or loss-of-function on β cells. Mice with loss of the paternal *Kcnq1* allele display lower β cell mass and glucose intolerance. This effect was reported to be independent of Kcnq1 itself and explained by the loss of the long non-coding RNA (lncRNA) *Kcnq1ot1* located in *Kcnq1* intronic region, which controls the expression of the cell cycle inhibitor *Cdkn1c* situated in the same locus [23]. *KCNQ1* −*/*− hESCs generated by an indel mutation can derive SC-β with no apparent differentiation defects, but these display impaired insulin secretion [208]. Recently, a missense mutation (R397W) in KCNQ1 was associated with neonatal diabetes in humans, and hESCs edited to carry the same mutation showed abnormal electrical activity and hypersecretion of insulin. However, prolonged culture of KCNQ1-R397W SC-β resulted in increased cell death and impaired insulin secretion [219]. A recent study found physical proximity between a T2D-risk SNP that falls within an enhancer located in exon 3 of *KCNQ1* and the *INS* promoter in human β cells by chromosome conformation capture assays. A 2.6 Kb region flanking this site was deleted in hESC and SC-β were derived with no apparent disparities in differentiation, but mutant cells showed decreased transcription of *INS* and *CDKN1C* [220]. While the association of KCNQ1 and glucose homeostasis is still enigmatic, it seems that multiple players beyond KCNQ1 might mediate the effects that explain the risk association.

- *SLC30A8*

The Zn^2+^ transporter encoded by this gene is responsible for the transport of Zn^2+^ into insulin granules [221]. The R325W hypomorphic missense variant of SLC30A8 was identified in the first wave of T2D-GWAS [188] and was found to protect against T2D development. Subsequent studies found other missense or protein-truncating variants of *SLC30A8* that suggest that haploinsufficiency protects against T2D [222]. There are conflicting reports on the consequences of Slc30a8 loss of function for glucose tolerance in mice [223,224,225]. Studies in human subjects that are carriers of the SLC30A8-W325 or a truncated variant showed increased insulin secretion after an oral glucose challenge [226]. These variants were introduced in an iPSC line by CRISPR-Cas9 genome editing, and allele-specific expression in SC-β determined that protective variants were hypomorphic. The authors also reported lower *INS* expression in cells carrying *SLC30A8* variants, which might suggest an effect on differentiation [226]. A recent study using a hESC *SLC30A8* −*/*− line reported improved GSIS in SC-β in vitro, and in vivo after transplantation. The authors also reported improved functional maturation and protection against death induced by metabolic stress [227]. Overall, the link between SLC30A8 loss-of-function and T2D is poorly understood mechanistically. The differences observed in mouse models and SC-β cells will surely lead to more research focused on unraveling the role of Zn^2+^ in human β cell function.

- *TCF7L2*

Variants in this gene locus are arguably the most potent T2D-risk polymorphisms identified by GWAS [188]. The gene encodes a transcription factor that acts as an effector of the Wnt signaling pathway [228]. The mechanism that associates this locus to disease risk is still enigmatic, despite being one of the most studied genes [229]. *TCF7L2* risk variants are found in non-coding regions, and these were associated with open chromatin sites in human islets. Enhancer activity for the lead SNP was shown to be increased by the risk variant [230]. Expression of *TCF7L2* is increased in T2D islets and to a larger extent in carriers of the risk allele [231]. Studies in mice have found that Tcfl7l2 loss-of-function in β cells has a detrimental effect on β cell mass and function [232,233]. Other studies argue that Tcf7l2 in non-β cell tissues is the main contributor to the altered glucose homeostasis phenotype observed in Tcf7l2-overexpressing mice [234]. TCF7L2 role during β cell development was studied by Weng and colleagues using hESCs [235]. *TCF7L2* is expressed at the PSC stage and in a second wave during PF and PP stages. During this second wave, a stage-specific enhancer was identified in the same LD block with risk SNPs, and deletion of this region resulted in decreased expression of *TCF7L2* and improved differentiation of endocrine precursors [235].

Beyond some of the aforementioned studies describing a single locus, there are others that used multi-omic approaches to survey T2D-GWAS loci. Nguyen and colleagues used an array of iPSC lines generated from unrelated individuals that were differentiated to PPs and performed transcriptomic (scRNA-seq) and chromatin accessibility (snATAC-seq) assays. Results were integrated with T2D-risk variants [236]. Another study performed scRNA-seq during all the steps of differentiation of hESCs toward β cells, generating a transcriptomic atlas of SC-β differentiation and paying special attention to the enrichment of genes linked to T2D-GWAS [235]. Epigenomic maps consisting of chromatin accessibility, histone marks and 3D chromatin architecture from hESC-derived progenitors, at multiple steps are also recently available [237,238].

### 3.3. Type 1 Diabetes (T1D)

T1D is a complex disease caused by autoimmune destruction of β cells. The strong genetic component of T1D [241] has been surveyed by GWAS [242,243,244,245]. The HLA region represents approximately half of the familial aggregation of T1D [246]. Other loci strongly associated with T1D are *INS*, *PTPN22*, *CTLA4* and *IL2RA* [243]. Most of the T1D-risk SNPs fall in non-coding regions (intronic or intergenic), with a few exceptions such as the R620W missense mutation in *PTPN22*, which is frequent in European populations [247]. Rare missense and frameshift mutations have also been identified by fine-mapping of the >50 loci associated with T1D [248], including protective variants [249]. At the cellular level, these risk variants may impact the function of CD8+ T cells, antigen presenting cells (APCs), other T1D-relevant immune cells or a combination of these. In addition, variants might alter normal β cell function or their response to cytokines. Recent studies also point to the potential role of some of these variants in acinar and duct cells of the pancreas [245].

The use of isogenic hESC platforms and patient-derived iPSCs to survey the genetics of T1D at multiple cellular levels has been suggested in the literature [250,251,252]. Studies with patient-derived stem cells to model T1D include examples such as: the use of iPSC-derived SC-β to evaluate their response to cytokines [253,254], generation of iPSC-derived APCs to model their interaction with T cells [255] or complex autologous platforms with iPSC-derived SC-β and immune cells from the same patients [256]. A fine example of the potential of stem cells to model T1D is illustrated in a study where patient-derived iPSCs were used to generate SC-β, macrophages, dendritic cells and endothelial cells. These were used in combination with T cell avatars from the same donor [257].

Since the first demonstrations of the functional capacity of SC-β generated from T1D patient-derived iPSCs [104,106], most of the research in the field has been focused on the potential clinical application of stem cells for cell replacement therapy [258]. Autologous systems have certain limitations, such as the cost [259], the risk associated with de-novo generation of immunogenic epitopes during reprogramming [260] or teratoma formation from residual undifferentiated cells [141]. Recent reports describe the use of immune-evasive SC-β organoids: immunotolerance was achieved by PD-L1 overexpression [261].

## 4. Classification of the Genetic Drivers of β Cell Dysfunction

**Figure 3:**
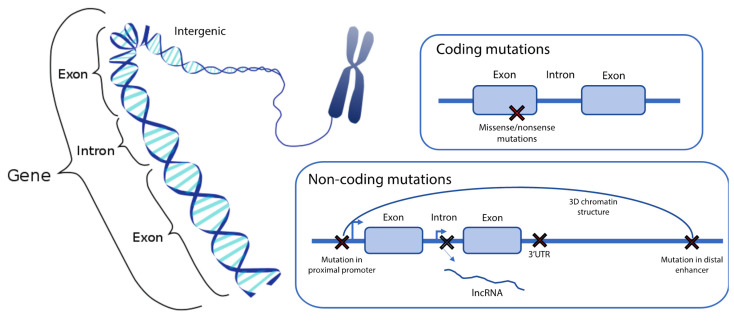
Schematic representation of genetic variants caused by mutations in coding/non-coding regions of the genome. Adapted DNA diagram (CC BY-SA 4.0 license, Smedib).

We can classify the genetic variants by protein coding and non-coding, and these are further subdivided as summarized in Figure 3.

### 4.1. Protein Coding Variants

Most of the genetic drivers of monogenic diabetes are associated with mutations in the protein coding sequence of factors important for pancreas development and/or β cell function. These can be missense mutations, such as INS-C96Y [122] or HNF1B-R165H [134], associated with neonatal diabetes and MODY, respectively, as well as, nonsense mutations resulting in truncated proteins—*RFX6* [262] or *GCK* [263] variants—or repeat insertions such as GAA trinucleotide repeat insertion in exon 1 of *FXN*, associated with Friedreich’s ataxia [139].

Coding variants identified in T2D-GWAS include the protective missense (Q325W) and nonsense (R138*) mutations in *SLC30A8* [222], and other low frequency (<5%) or rare (<0.5%) coding mutations identified by exome sequencing in genes such as *TBC1D30*, *KANK1*, *PAM* or *PPIP5K2*, which have been associated with altered insulin processing and secretion [190] or a *CCND2* coding variant associated with increased expression, and T2D-risk reduction by half [9], among many others [264]. The overall contribution to the disease risk of common variants (mostly non-coding) and low-frequency/rare variants in coding sequences is debated elsewhere [8,265,266].

### 4.2. Non-Coding Variants

Genetic variants in the non-coding regions of the genome have been described to play an important role in different human diseases [267]. Most of the SNPs identified in T1D and T2D-GWAS map to non-coding regions of the genome, and there are also examples of non-coding variants leading to monogenic forms of diabetes [268,269,270]. These variants usually lead to altered transcriptional regulation [78,79,271]. GWAS-identified T2D-risk SNPs are particularly enriched in β cell regulatory elements [71]. A catalog of cell-specific *cis*-regulatory elements—from 222 different cell types—mapped disease-related likely causal variants. β cell-specific regulatory elements ranked highest for T2D, while variants enriched in CD4+/CD8+ T-cell-specific elements were more common for T1D-risk variants [272]. Some examples of these genetic variants are classified and described below.

#### 4.2.1. Promoter

Non-coding variants that fall near transcriptional start sites have significantly larger eQTL effects compared to those found in distal enhancers [273]. Some variants with substantial expression effects are associated with MODY, such as promoter mutations in *GCK*, *HNF1A* or *HNF4A* [268,274,275,276,277,278,279,280] or neonatal diabetes with SNPs in the CC element of the *INS* promoter [281,282]. GWAS-identified SNPs in loci associated with altered promoter activity include *ARAP1* [283], *G6PC2* [284,285] or the T1D-candidate gene *CTSH* [286]. Promoters are necessary for transcription initiation, but they are not sufficient to direct tissue-specific dynamic gene expression. Additional *cis*-regulatory elements are required for complete transcriptional regulation.

#### 4.2.2. Enhancer

Enhancers are necessary for robust and correct spatiotemporal gene expression. Genome-wide survey of open chromatin accessibility by DNase-seq, ATAC-seq and histone modification ChIP-seq has identified unique regulatory elements that reveal tissue-specific features [272,287]. Studies integrated with T2D-GWAS found risk signals enriched at enhancers active in islets [72,230,288,289] or in hESC-derived progenitors [237]. Several studies have attributed gene expression effects and diabetes-GWAS signals to causal SNPs in enhancer regions. *TCF7L2* risk variant rs7903146 maps to an islet enhancer region and is associated with increased activity [230]. Deletion of this enhancer region in hESC leads to decreased *TCF7L2* expression specifically in the PF and PP stages [235]. Gaulton and colleagues found enrichment of T2D-GWAS signals in FOXA2-binding enhancer sites from islet and liver ChIP. They described how the risk allele of rs10830963 leads to increased expression of *MTNR1B* by creating a NEUROD1-binding site in an enhancer region associated with FOXA2 [290]. The opposite effect was found for the risk variant of rs58692659, which disturbs a NEUROD1-binding enhancer, leading to decreased *ZFAND3* expression [71]. More examples of β cell enhancer-mediated effects in GWAS loci include *CAMK1D* [35], *ADYC5* [291], *C2CD4A* [292], *DGKB* [78] or *CLEC16A* [293,294], among others. By epigenomic mapping of hESC differentiation, Geusz and colleagues found progenitor-specific enhancers at the *LAMA1* and *CRB2* loci. CRISPR-mediated deletion of these enhancer regions identified critical, step-specific regulation of the expression of these genes. Indeed, T2D-risk variants were found increased in these regions [237]. While effects derived from common enhancer variants are relatively small, there are other rare variants with profound impact. Using hESC-derived PPs, Weedon and collaborators generated an annotated map of enhancer marks and regulatory elements binding FOXA2, PDX1, GATA, HNF1B and ONECUT, at this stage of development. These led to the identification of a distal *PTF1A* enhancer, not found in other adult or embryonic tissues. Recessive mutations in this enhancer were found in previously unexplained cases of pancreatic agenesis [270]. Likewise, another study described an SNP in an enhancer that alters *GATA6* expression in iPSC-derived PPs, and this is described to modify disease penetrance in patients with heterozygous *GATA6* mutations [269].

#### 4.2.3. Three-Dimensional Chromatin Structure

The distance between gene-coding regions and their regulatory elements can be as far as 2–3 Mb [295,296]. Interaction between target genes and distal regulatory elements is mediated by chromatin loops. The 3D structure of chromatin results from the interplay between promoters, enhancers and insulators, and is regulated by elements such as CTCF, cohexin and Polycomb complexes, along with other factors that confer spatiotemporal specificity [297,298,299]. Chromosome conformation capture assays provide a picture of these chromatin interactions [300]. Genome-wide profiling of chromosome architecture in human islets [75,301], sorted populations of the pancreas [302], EndoC-βH1 cells [61,303] and hESC-derived progenitors and SC-β [220,237] provides additional tools for the interrogation of GWAS signals. T2D-risk SNPs in the *STARD10* locus fall into a CTCF-flanked enhancer and this was found to interact with the *FCHSD2* promoter in human islets. Both genes were found to be important for insulin secretion, and risk variants exerted a negative expression effect on both [304,305]. Two recent independent reports have identified long-range contact between the *INS* locus in chromosome 11 and *KRTAP5-6* [303] or *KCNQ1* locus [220], both containing T2D-GWAS signals.

#### 4.2.4. Non-Coding RNA (ncRNA)

Long non-coding RNAs (lncRNAs) and microRNAs (miRNAs) are transcripts that lack protein-coding potential but have biological roles through chromatin remodeling, regulation of splicing, imprinting induction, translation regulation and modulation of mRNA stability. Their impact on β cell function and development is reviewed elsewhere [306,307,308]. β cell-specific expression of lncRNAs [69] and miRNAs [309] is dynamically regulated and altered in T2D. Some of the risk variants identified in GWAS have been associated with causal effects mediated by ncRNAs: these include SNPs in the lncRNA *ANRIL* locus which impacts *CDKN2A/B* expression [310] or in the *CDKN1C*-associated lncRNA *KCNQ1OT1* [23,311]. The biological effect of miRNAs is mediated by direct coupling to their target mRNA, most frequently at 3′-untranslated regions (3′UTR) or near the stop codon. Some studies have identified risk variants that alter predicted miRNA-binding sites [309,312,313] in genes such as *SLC30A8*, *INSR* or *LPL*, among others. However, few of the predicted effects have been validated or further studied.

#### 4.2.5. Other Non-Coding Variants

Association of genetic variants with disease risk has been frequently attributed to transcripts through expression QTLs (eQTLs), and chromatin accessibility QTLs (caQTLs). However, there are additional mechanisms that might explain disease risk. One of these is altered splicing, which can result from SNPs and impact disease risk of complex traits independently from effects on gene expression [314]. An atlas of splicing QTLs (sQTLs) across human islets was recently generated, and this constitutes an additional layer to explore the genetic basis of T2D risk [70]. The authors found sQTLs in previously reported T2D-risk loci but also found small-effect T2D-associations in new candidate genes, such as an exon-skipping variant of *ERO1B*. Prior to this report, others have also associated T2D-risk SNPs with splicing effects: *HNF1A* [315], *CDKAL1* [207] or *G6PC2* [285].

DNA methylation can also be altered as a result of genetic variants: epigenome-wide approaches on human islets have identified hypo- and hyper-methylated regions in T2D, some of these mapping to GWAS loci such as *ADYC5* or *KCNQ1* [316,317]. T2D-risk SNPs at potential sites of methylation (CpG dinucleotides) have also been mapped [318,319]. There are other underexplored DNA modifications such as 5-hydroxymethylcytosine (5hmC) that have an impact in chromatin architecture and gene expression. 5hmC is dynamically regulated during differentiation of SC-β from hESCs [320,321] but the impact of genetic variants on 5hmC remains to be explored.

Lastly, RNA modifications can impact RNA stability and translation. Out of the hundreds of different RNA modifications described, *N*^6^-methyladenosine (m^6^A) is the most widely characterized [322]. Most m^6^A modifications map into non-coding regions (3′UTR, 5′UTR) but are also enriched in the coding sequence near stop codons. Transcriptome-wide mapping of m^6^A-QTLs in lymphoblastoid cell lines found association with blood and immune-related traits, such as T1D [323]. SNPs that disturb the m^6^A consensus motif or, more frequently, changes in RNA-binding protein target sites were putatively causal for m^6^A variation. The impact of genetic variants that alter m^6^A in T2D-associated loci has only been surveyed in silico [324].

## 5. Classification of Processes Associated with β Cell Dysfunction

The last section of this review provides a brief classification of four processes associated with β cell dysfunction. Each section summarizes approaches to investigate the contribution of genetic variants, focusing on the use of stem cell differentiation protocols.

### 5.1. Pancreas Development

The use of stem cells and differentiation protocols enables the possibility of modeling human pancreas development. As already described in this review, many of the genetic variants responsible for monogenic forms of diabetes play a critical role during pancreas development. Some of the common variants influencing polygenic disease risk are also potentially involved in pancreas development, as seen from multi-omic analyses of hESC differentiation toward SC-β [235,237]. The possibility of modeling the effect of single genetic variants with the use of isogenic controls can facilitate personalized medicine approaches. There are examples of deleterious variants that are only found in specific populations, which might play a role in pancreas development. One of these examples is the rs137853240 variant leading to a missense G319S mutation in *HNF1A*, only found in Ontario Oji-Cree (allele frequency 0.2), one of the populations with the highest T2D incidence in the world [315,325]. Modeling the effect of this or other genetic variants implicated in pancreas development can help guide us to better treatments for at-risk patients.

### 5.2. β Cell Mass and Adaptive Proliferation

Studies in rodents have shown that changes in β cell mass are pivotal in T2D progression [326]. Insulinemia and glycemic levels are directly related to total β cell mass in rodent experiments where other variables can be controlled. Studies of β cell mass in humans are particularly challenging, and since β cell mass can only be surveyed in post mortem samples, we lack longitudinal studies. Still, there is evidence that knowledge acquired from rodent studies can be translated to the pathophysiology of humans [327]. Post-mortem studies have shown that diabetes onset is associated with a decrease of β cell mass in obese and lean human subjects [328]. These studies also highlight the heterogeneity of β cell mass—up to 10-fold—found across individuals [329]. β cell mass-focused approaches in humans are inherently limited by the fact that observations are transversal in time and cannot provide information on the dynamic changes that might have occurred. Advances of in vivo imaging techniques allowing β cell mass determination could overcome these limitations, but until then, models that allow longitudinal measurements are attractive for researchers that study β cell mass. Beyond the use of mice, translucent zebrafish are interesting since they allow continuous observation [39,40,237]. Total pancreas mass, and subsequently β cell mass, is determined by the size and proliferation of the pancreas progenitor pool [330]. For these reasons, studies that employ stem cell platforms represent an attractive approach to investigate the genetic and epigenetic components of “β cell mass endowment”. On the other hand, modeling adaptive proliferation of adult β cells with SC-β has potential advantages but also some weaknesses that should be addressed. Basal proliferation of SC-β is usually defined as “low” but is still much higher than that of native adult human β cells (1% vs. 0.2–0.5%) [331,332]. It is possible that future improvements in differentiation protocols will enhance SC-β maturity bringing the proliferation rate close to that of adult β cells. Indeed, this tradeoff between β cell proliferation and maturity is reported in SC-β and murine β cells [333,334,335]. SC-β platforms for screening approaches might represent a cost-effective alternative when compared with human islets and will also bypass the problems associated with wide heterogeneity in human samples. A screening of Zn^2+^-binding prodrugs targeting β cell proliferation is one example of this [331]. Other screening approaches based on scRNA-seq of SC-β clusters have identified a sub-population of proliferating SC-β, characterized by LIF-pathway upregulation. The authors further confirmed that LIF is able to increase the proliferation of native human β cells [336].

### 5.3. β Cell Function

Stem cell-based approaches interrogating the influence of genetic variants on β cell function have been extensively described in this review. This approach has proven useful for understanding monogenic drivers of disease and assessing the function of candidate genes with gain- or loss-of-function studies [117,208]. Despite years of advances, SC-β are still not a match for islets from cadaveric donors in terms of mature functionality. SC-β mediated approaches are attractive to explore the molecular effects of risk or protective variants [226]. However, modeling the functional consequences (i.e., insulin secretion) of small-effect risk variants, in a still-immature model, seems a futile approach. However, other screening strategies that were initially aimed at improving SC-β differentiation protocols are also uncovering pathways that are important for the function of native β cells [109,111,113,337].

### 5.4. Response to Stress

β cells are particularly susceptible to stress (cytokines, ER stress, oxidative stress), which can lead to functional demise, dedifferentiation or even cell death. Thus stress-mediated β cell dysfunction is reported to play an important role in diabetes pathophysiology [338,339,340]. Stem cell platforms have been used to model these stress responses. SC-β generated from T1D-patient fibroblast iPSCs respond to cytokines and other chemical stressors in a manner similar to that of β cells [104]. Others have also probed the response of iPSC-derived SC-β to cytokines [253,254] or study their interaction with immune cells in complex isogenic systems [257]. The use of gene-editing techniques on iPSC/hESC to study T1D-predisposing SNPs in response to cytokines or interaction with immune cells is an approach that has already been considered [251]. Several other studies have focused on the influence of genetic variants on β cell ER stress. SC-β derived from Wolfram syndrome patients display ER stress and dysfunction and a chemical chaperone is able to ameliorate stress and restore function [161]. *INS* or *MANF* mutations also cause ER stress and β cell dysfunction and increased cell death in response to chemical stressors such as brefeldin A [138,157]. *TRMT10A* deficiency induces oxidative stress and apoptosis in SC-β derived from iPSCs [163]. Lastly, SC-β derived from *GLIS3* −/− hESC display impaired differentiation and significant cell death. A chemical screening identified a TGFBR1 inhibitor capable of reducing cell death [170].

## 6. Limitations

While research using stem cell-derived SC-β allows for the modelling of human development and overcomes some of the limitations of other research platforms, stem cell-based approaches also have their own limitations. Some of them, such as incomplete maturity of SC-β, were already discussed in the previous section. β cells are sensors of the nutritional status of the organism but also integrate signals from multiple other cell types. These include local signals derived from the vasculature or other endocrine cells to systemic signals from gut-derived incretins, adipokines, liver or bone signals, among others [341]. These signals are important not only for the normal functioning of mature β cells but also for normal development [342,343]. Some of the effects derived from these interactions might be missed in screening approaches that focus on the use of SC-β alone. Other important factors contributing to disease risk, such as β cell-derived effects from intrauterine epigenetic programming [344,345] are difficult to model with stem cell platforms [346]. Other limitations are derived from the genetic variability and differentiation potential of iPSCs [142]: comparisons of iPSCs derived from different donors are limited by this and isogenic controls are ideal when possible [347]. Cultured PSCs frequently acquire genomic abnormalities during extended culture [348]: proper culture conditions and periodic assessment of genomic integrity and pluripotency are laborious but needed to maintain high-quality cultures. Lastly, maintenance of pluripotent lines and the reagents used for differentiation experiments are costly. Fortunately, there have been recent advances in reducing expenses by new formulations or by reducing sample size in high-throughput experiments [331,349]. Other limitations such as ethical or legal issues or limitations in stem cell-based therapy are beyond the scope of this review and discussed in depth elsewhere [350,351].

## 7. Conclusions

Studies linking genetic information to pathophysiology will bring us closer to personalized medicine. Therefore, identifying genetic predictors of β dysfunction and dissecting the role of the molecular effectors will enable the introduction of novel targeted T2D therapies and prevention strategies. Stem cell research is a promising avenue to expand our knowledge of the genetic basis of β cell dysfunction.

## Figures and Tables

**Table 1:** Summary of stem cell lines used to study genetic variants and their impact in endocrine development and β cell function. Variants listed are loss-of-function/hypomorphic unless stated otherwise.

Genetic Variant	Cell Lines	Phenotype	References
*ABCC8* V187D	Patient-derived iPSC and isogenic controls	Increased insulin secretion in low glucose, increased proliferation	[166]
*ARX* −*/Y*	hESC	No α cells and differentiation shifted toward δ cells	[117]
*CACNA1A* −*/*−	hESC	Normal differentiation, reduced intracellular Ca^2+^ levels, impaired GSIS	[146]
*CDKAL1* −*/*−	hESC	Normal differentiation, impaired GSIS, susceptibility to ER stress	[208,209]
*FOXA1* −*/*−	hESC	Dispensable for PP generation	[239]
*FOXA2 +/*−, −*/*−	Patient-derived iPSC and healthy controls, hESC	Impaired generation of PPs	[164,239,240]
*GATA4 +/*−	hESC	*GATA4* dosage influences the phenotype in *GATA6 +/*−	[168]
*GATA6* −*/*−, R456C, Val204fs	hESC	Impaired endoderm differentiation (−/−) or formation of PP stage (+/−)	[168]
*GLIS3* −/−	hESC	Impaired differentiation of PPs and Eps, cell death	[170]
*HES1* −/−	hESC	Accelerated endocrine differentiation	[117]
*HNF1 +/*−, −/−, Pro291fs, R200Q	hESC and patient-derived iPSC with isogenic controls	Differentiation biased toward α cells, impaired GSIS, altered insulin:C-peptide stoichiometry	[145,146,148]
*HNF1B* R177*, S148L	Patient-derived iPSC and family controls	Hypomorphic variants, decreased *PAX6* expression	[153,154]
*HNF4A* Ile271fs, Q268*	Patient-derived iPSC and family controls	Altered differentiation with decreased *HNF1A* and *PDX1* expression	[149,150,151]
*INS* C96R, *INS^M1I/M1I^*	Patient-derived iPSC and isogenic controls	C96R: ER stress, reduced function*INS^M1I/M1I^*: total absence of insulin	[157,158]
*KCNJ11* −*/*−	hESC	Normal differentiation, impaired GSIS	[208]
*KCNQ1* −*/*−, R397W	hESC	−/−: Normal differentiation, impaired GSIS R397W: hypersecretion of insulin, followed by cell death and functional demise	[208,219]
*KCNQ1 ΔEnhancer*	hESC	Decreased *INS* and *CDKN1C* expression	[220]
*NEUROD1* −/−	hESC	Impaired endocrine differentiation	[169]
*NEUROG3* −*/*−, *+/*−	hESC	Abolished/highly impaired generation of endocrine cells	[117,175]
*NEUROG3*-gain-of-function	hESC	Accelerated endocrine differentiation	[117]
*NOTCH1*-gain-of-function (N1ICD)	hESC	Abolished endocrine differentiation	[117]
*ONECUT1* −*/*−, E231*, E231D	Patient-derived iPSC and hESC	Altered NKX6.1 and NKX6.2 during PP stage	[136]
*PAX4 −/−*	hESC	Differentiation biased toward α cells	[146]
*PDX1 −/−*, P33T, C18	Patient-derived iPSC and healthy control. hESCs	Reduced differentiation efficiency.−/−: abolished differentiation	[13]
*RFX6 −/−*	hESC	Abolished endocrine differentiation	[117]
*SLC30A8 −/−*, R138*, Ser38fs	hESC	Hypomorphic variants, improved GSIS	[226,227]
*STAT3 K392R* (activating mutation)	Patient-derived iPSC and isogenic controls	Premature differentiation, biased toward α cells, upregulation of NEUROG3 and targets	[165]
*SYT13 −/−*	hESC	Normal differentiation, impaired GSIS	[146]
*TCF7L2 ΔEnhancer*	hESC	Improved differentiation of EPs	[235]
*TRMT10A* R127*, E27*	Patient-derived iPSC and healthy controls	Oxidative stress and apoptosis	[163]
*WFS1* various mutations	Patient-derived iPSC and healthy or isogenic controls	Normal differentiation, ER stress, decreased insulin content	[161,162]
*YIPF5* −/− and I98S	hESC and patient-derived iPSC with isogenic controls	Proinsulin retention in the ER, susceptible to ER stress-induced death	[159]
*ZNF808 −/−*	hESC	Inappropriate specification of cell fate, loss of pancreatic identity	[128]

## Data Availability

Not applicable.
